# Relationship between the platelet-to-lymphocyte ratio and in-hospital mortality of ischemic stroke patients in the intensive care unit

**DOI:** 10.3389/fnagi.2025.1607332

**Published:** 2025-08-12

**Authors:** Guohua Liu, Ya Zhou, Hao Ding, Lin Chen, Lan Chen, Sufang Yang

**Affiliations:** ^1^Department of Pharmacy, Affiliated Hospital of Jinggangshan University, Ji’an, Jiangxi, China; ^2^Department of Pharmacy, People’s Hospital of Ningxiang City Affiliated to Hunan University of Chinese Medicine, Changsha, Hunan, China; ^3^Department of Clinical Medicine, Baoying People’s Hospital, Baoying Clinical Medical College of Yangzhou University, Yangzhou, Jiangsu, China; ^4^Department of Neurology, Affiliated Hospital of Jinggangshan University, Ji’an, Jiangxi, China

**Keywords:** platelet-to-lymphocyte ratio, ischemic stroke, in-hospital mortality, relationship, intensive care unit

## Abstract

**Background:**

The relationship between the platelet-to-lymphocyte ratio (PLR) and the prognosis of patients with ischemic stroke was unclear.

**Objective:**

This study aimed to explore the correlation between PLR levels and in-hospital mortality in ischemic stroke patients admitted to the intensive care unit (ICU).

**Methods:**

A retrospective cohort study was conducted using data from the MIMIC-IV database. Demographic and clinical data of all participants were collected, and the study outcome was in-hospital mortality. Patients were divided into three groups based on the tertiles of PLR: low PLR group (PLR < 0.88), intermediate PLR group (0.88 ≤ PLR < 1.73), and high PLR group (PLR ≥ 1.73). Multivariable-adjusted logistic regression analysis, curve fitting, interaction analysis, and threshold analysis were performed to evaluate the relationship between PLR levels and in-hospital mortality in ischemic stroke patients in the ICU.

**Results:**

A total of 1,002 critically ill patients with ischemic stroke were included, with an average PLR level of 1.88 ± 2.34. The overall in-hospital mortality rate was 12.48%, with mortality rates of 7.38% in the low PLR group, 8.96% in the intermediate PLR group, and 21.15% in the high PLR group. A non-linear J-shaped relationship was found between PLR and in-hospital mortality. The study found that when the PLR value was less than 4.21, there was a positive correlation between PLR and in-hospital mortality. In the subgroup analysis, no statistically significant interactions were found among the subgroups.

**Conclusion:**

In the ICU setting, PLR levels were independently associated with in-hospital mortality in critically ill patients with ischemic stroke. When PLR was less than 4.21, this emphasized the importance of close monitoring by ICU physicians.

## Introduction

Ischemic stroke is one of the leading causes of death and long-term disability worldwide, especially in intensive care units (ICUs), where the condition of patients is more severe. Prognostic assessment is crucial for optimizing treatment strategies and improving patient outcomes (Zhang et al., 2024). In recent years, many biological markers related to the prognosis of ischemic stroke, such as the triglyceride–glucose index, stress hyperglycemia ratio (Chen et al., 2022), and neutrophil–lymphocyte ratio (Gong et al., 2019), have been studied and confirmed. However, studies on the relationship between the platelet–lymphocyte ratio (PLR) and in-hospital mortality in ischemic stroke patients in the ICU are relatively limited.

After ischemic stroke occurs, the body is in a state of stress, triggering a series of inflammatory responses (Koutsaliaris et al., 2022). Platelets play an important role in the inflammatory process, as they can release a variety of inflammatory mediators and participate in the inflammatory cascade (Couldwell and Machlus, 2019). Lymphocytes, on the other hand, are an important part of the immune system, and changes in their numbers reflect the state of the body’s immune function (Chreshnev et al., 2004). An elevated PLR might indicate enhanced inflammatory responses and suppressed immune function, and this imbalance could be detrimental to the recovery of patients, thereby increasing in-hospital mortality. Numerous studies have shown that the PLR is an independent prognostic predictor of ischemic stroke (Zhang et al., 2023; Ma et al., 2022). Altintas et al. (2016) reported that in acute ischemic stroke (AIS) patients, an elevated PLR was associated with infarct size and a poor prognosis. Yan et al. (2021) conducted a meta-analysis and reported that patients with a PLR > 150 had an in-hospital mortality rate 2.35 times higher than those with a PLR ≤ 150; high PLR levels were also associated with an increased risk of early neurological deterioration. One study showed that PLR levels were independently correlated with increased National Institutes of Health Stroke Scale (NIHSS) scores, suggesting that high PLR levels might indicate more severe neurological impairment (Kömürcü et al., 2020). However, some studies have failed to find a correlation between the PLR and the prognosis of ischemic stroke patients (Li, 2021; Ferro et al., 2021). The reason for the lack of correlation might be due to differences in the study populations, such as the failure to target patients in the ICU.

Given the conflicting reports in the current literature, we chose to focus on ischemic stroke patients in the ICU for the first time to clarify the association between PLR levels and in-hospital mortality and to explore the specific ranges of the PLR in relation to in-hospital mortality. This research is expected to provide new ideas and methods for the clinical management of such patients.

## Material and methods

### Data source

The data used in this study came from MIMIC-IV 3.0 (Medical Information Mart for Intensive Care), a large, open, intensive care clinical research database. The data collected included patient demographic information, common laboratory indicators at admission, comorbidities, medication use, and survival prognosis. A member of this study team, who had been trained and certified (certification number 67219293), was responsible for accessing and extracting data from the database. The MIMIC database followed the review board protocol, and all patient personal information was deidentified via random codes to identify specific patients. The Ethics Committee of the Affiliated Hospital of Jinggangshan University approved this study to be exempt from informed consent and ethical review requirements.

### Inclusion and exclusion criteria

Inclusion criteria: Patients diagnosed with ischemic stroke were identified using the International Classification of Diseases (ICD-9 and ICD-10) codes. Exclusion criteria: (1) Patients who were not admitted to the ICU for the first time and had their first hospital admission; (2) Patients under the age of 18; (3) Patients without ICU admission data; (4) Patients with malignant tumors, leukemia, myelodysplastic syndromes, systemic lupus erythematosus, or rheumatoid arthritis; (5) Patients who had undergone splenectomy; (6) Patients without complete records of platelet and lymphocyte counts at the first ICU admission. A total of 5,253 patients were initially included, and ultimately, 1,002 patients with ischemic stroke were included in the study analysis according to the inclusion and exclusion criteria.

### Data extraction

The research group used structured query language (SQL) and PostgreSQL 15 tools to extract and manage the data. The extracted data included the number of admissions, age, sex, past medical history, and laboratory indicators of stroke patients. The past medical history included hypertension, diabetes, malignancies, acute renal failure, pneumonia, and sepsis. The laboratory indicators included hemoglobin, potassium, sodium, PLR, white blood cells, blood glucose, creatinine, and prothrombin time. The main outcome of the study was whether the patient died during the hospital stay.

### Statistical analysis

**FIGURE 1:**
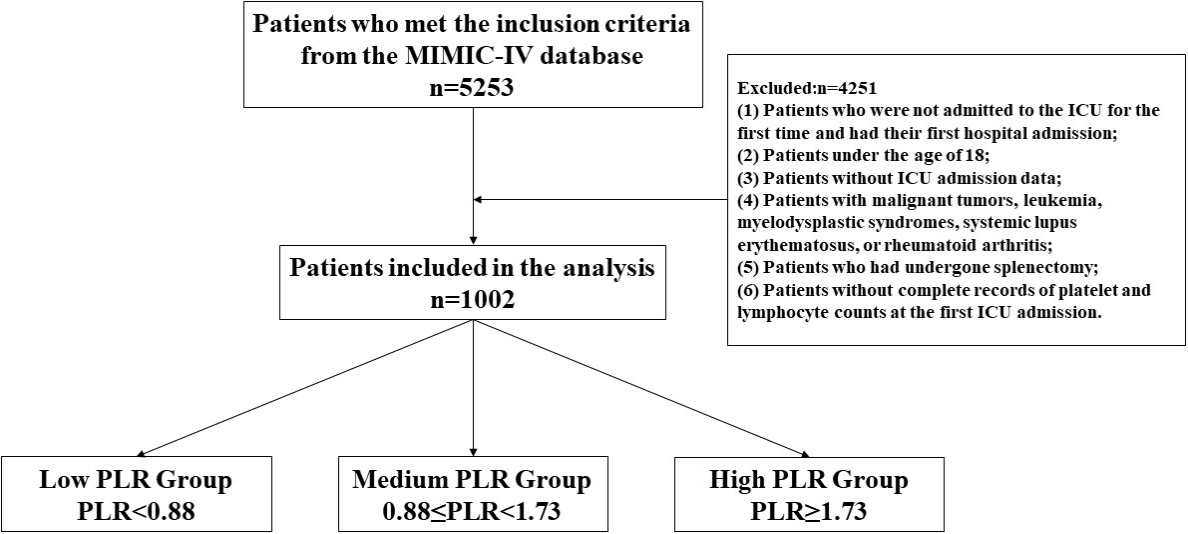
Flowchart of patient selection. PLR, platelet-to-lymphocyte ratio.

The study group was divided on the basis of whether death occurred, and the statistical significance of differences between variables was assessed. Continuous variables that followed a normal distribution are expressed as the means ± standard deviations and were analyzed via *t*-tests; variables that did not follow a normal distribution are expressed as interquartile ranges (IQRs) and were analyzed via the Wilcoxon rank-sum test. Categorical variables are expressed as percentages and were compared via the chi-square test. All the statistical analyses were performed via R software (version 4.3.0) and Zstats v1.0 (www.zstats.net), with a two-sided *p*-value of less than 0.05 considered statistically significant. The study methods and procedures are detailed in Figure 1.

## Results

### Baseline characteristics of the study population

We included 1,002 patients with ischemic stroke in the intensive care unit based on the inclusion and exclusion criteria. Among them, 125 patients died in the hospital, accounting for 12.48%. The patients who died in the hospital had a lower prevalence of hypertension; a higher prevalence of acute renal failure, pneumonia, and sepsis; elevated PLR, white blood cell count, serum sodium level, blood glucose value, prothrombin time, and serum creatinine level; decreased serum calcium level; and required more mechanical ventilation (see Table 1). We divided the PLR into tertiles and found that changes in PLR levels were associated with white blood cell count, hemoglobin level, blood glucose, serum creatinine level, as well as the presence of heart failure, acute renal failure, pneumonia, hyperlipidemia, chronic obstructive pulmonary disease (COPD), sepsis, and mechanical ventilation (see Table 2).

**TABLE 1:** Baseline characteristics of the study population according to the in-hospital mortality status of patients with cerebral infarction in the ICU.

Variables	Total (*n* = 1002)	Survivors (*n* = 877)	Non-survivors (*n* = 125)	*P*
Age, mean ± SD	65.09 ± 10.60	64.99 ± 10.50	65.82 ± 11.28	0.415
Gender, n (%)				0.275
Female	404 (40.32)	348 (39.68)	56 (44.80)	
Male	598 (59.68)	529 (60.32)	69 (55.20)	
BMI (kg/m^2^), mean ± SD	29.88 ± 5.54	29.87 ± 5.40	29.95 ± 6.42	0.892
WBC (K/uL), mean ± SD	12.82 ± 5.88	12.41 ± 5.44	15.70 ± 7.82	**<0.001**
Hemoglobin (g/dL), mean ± SD	10.67 ± 2.18	10.71 ± 2.16	10.40 ± 2.34	0.138
Sodium (mEq/L), mean ± SD	138.88 ± 4.66	138.71 ± 4.41	140.04 ± 6.05	**0.020**
Potassium (mEq/L), mean ± SD	4.29 ± 0.57	4.28 ± 0.55	4.35 ± 0.70	0.256
Calciumtotal (mg/dL), mean ± SD	8.45 ± 0.71	8.47 ± 0.69	8.28 ± 0.81	**0.011**
Glucose (mg/dL), mean ± SD	145.97 ± 60.63	142.36 ± 56.68	171.28 ± 79.01	**<0.001**
PT (sec), mean ± SD	15.46 ± 7.49	14.86 ± 6.16	19.71 ± 12.83	**<0.001**
Creatinine (mg/dL), mean ± SD	1.47 ± 1.42	1.40 ± 1.38	1.93 ± 1.65	**<0.001**
HT, n (%)	498 (49.70)	452 (51.54)	46 (36.80)	**0.002**
DM, n (%)	429 (42.81)	371 (42.30)	58 (46.40)	0.386
HF, n (%)	288 (28.74)	245 (27.94)	43 (34.40)	0.135
MI, n (%)	166 (16.57)	144 (16.42)	22 (17.60)	0.740
CKD, n (%)	228 (22.75)	192 (21.89)	36 (28.80)	0.085
ARF, n (%)	390 (38.92)	309 (35.23)	81 (64.80)	**<0.001**
Pneumonia, n (%)	216 (21.56)	166 (18.93)	50 (40.00)	**<0.001**
HLP, n (%)	566 (56.49)	503 (57.35)	63 (50.40)	0.142
COPD, n (%)	141 (14.07)	117 (13.34)	24 (19.20)	0.078
Sepsis, n (%)	482 (48.10)	383 (43.67)	99 (79.20)	**<0.001**
Ventilation, n (%)	762 (76.05)	654 (74.57)	108 (86.40)	**0.004**
Hospday (day), mean ± SD	13.56 ± 14.02	13.51 ± 13.40	13.89 ± 17.85	0.780
PLR, mean ± SD	1.88 ± 2.34	1.75 ± 2.18	2.81 ± 3.11	**<0.001**

WBC, white blood cell; BMI, body mass index; PT, prothrombin time; HT, hypertension; DM, diabetes mellitus; HF, heart failure; MI, myocardial infarction; HLP, hyperlipidemia; Hospday, length of hospital stay; CKD, chronic kidney disease; ARF, acute renal failure; Ventilation, mechanical ventilation; COPD, chronic obstructive pulmonary disease; PLR; platelet(105/ul)-to-lymphocyte(103/ul) ratio. Bold values indicate *P* < 0.05 and are statistically significant.

**TABLE 2:** Baseline characteristics of the study population according to PLR thresholds.

Variables	Total (*n* = 1002)	PLR < 0.88 (*n* = 325)	0.88 ≤ PLR < 1.73 (*n* = 346)	PLR ≥ 1.73 (*n* = 331)	*P*
Age, mean ± SD	65.09 ± 10.60	64.69 ± 10.35	64.67 ± 11.50	65.93 ± 9.81	0.216
Gender, n (%)					0.052
Female	404 (40.32)	120 (36.92)	133 (38.44)	151 (45.62)	
Male	598 (59.68)	205 (63.08)	213 (61.56)	180 (54.38)	
BMI (kg/m^2^), mean ± SD	29.88 ± 5.54	30.05 ± 5.61	29.48 ± 5.16	30.13 ± 5.83	0.246
WBC (K/uL), mean ± SD	12.82 ± 5.88	14.22 ± 6.22	11.88 ± 5.09	12.43 ± 6.08	**<0.001**
Hemoglobin (g/dL), mean ± SD	10.67 ± 2.18	10.37 ± 1.91	11.11 ± 2.27	10.51 ± 2.27	**<0.001**
Sodium (mEq/L), mean ± SD	138.88 ± 4.66	139.05 ± 4.29	139.02 ± 4.96	138.55 ± 4.69	0.303
Potassium (mEq/L), mean ± SD	4.29 ± 0.57	4.34 ± 0.56	4.26 ± 0.54	4.28 ± 0.62	0.183
Calciumtotal (mg/dL), mean ± SD	8.45 ± 0.71	8.37 ± 0.69	8.50 ± 0.68	8.47 ± 0.75	0.058
Glucose (mg/dL), mean ± SD	145.97 ± 60.63	135.55 ± 48.93	144.26 ± 63.75	157.98 ± 65.55	**<0.001**
PT (sec), mean ± SD	15.46 ± 7.49	15.31 ± 4.59	14.94 ± 5.93	16.17 ± 10.59	0.093
Creatinine (mg/dL), mean ± SD	1.47 ± 1.42	1.31 ± 1.20	1.40 ± 1.37	1.70 ± 1.63	**<0.001**
HT, n (%)	498 (49.70)	166 (51.08)	185 (53.47)	147 (44.41)	0.052
DM, n (%)	429 (42.81)	129 (39.69)	156 (45.09)	144 (43.50)	0.352
HF, n (%)	288 (28.74)	97 (29.85)	82 (23.70)	109 (32.93)	**0.026**
MI, n (%)	166 (16.57)	53 (16.31)	54 (15.61)	59 (17.82)	0.731
CKD, n (%)	228 (22.75)	64 (19.69)	73 (21.10)	91 (27.49)	**0.039**
ARF, n (%)	390 (38.92)	113 (34.77)	121 (34.97)	156 (47.13)	**<0.001**
Pneumonia, n (%)	216 (21.56)	47 (14.46)	74 (21.39)	95 (28.70)	**<0.001**
HLP, n (%)	566 (56.49)	199 (61.23)	212 (61.27)	155 (46.83)	**<0.001**
COPD, n (%)	141 (14.07)	37 (11.38)	42 (12.14)	62 (18.73)	**0.011**
Sepsis, n (%)	482 (48.10)	144 (44.31)	135 (39.02)	203 (61.33)	**<0.001**
Ventilation, n (%)	762 (76.05)	281 (86.46)	236 (68.21)	245 (74.02)	**<0.001**
Hospday, mean ± SD	13.56 ± 14.02	12.88 ± 12.73	12.86 ± 13.59	14.97 ± 15.52	0.084
Dead, n (%)	125 (12.48)	24 (7.38)	31 (8.96)	70 (21.15)	**<0.001**
PLR, mean ± SD	1.88 ± 2.34	0.61 ± 0.18	1.25 ± 0.24	3.79 ± 3.30	**<0.001**

WBC, white blood cell; BMI, body mass index; PT, prothrombin time; HT, hypertension; DM, diabetes mellitus; HF, heart failure; MI, myocardial infarction; HLP, hyperlipidemia; Hospday, length of hospital stay; CKD, chronic kidney disease; ARF, acute renal failure; Ventilation, mechanical ventilation; COPD, chronic obstructive pulmonary disease; PLR; platelet(105/ul)-to-lymphocyte(103/ul) ratio. Bold values indicate *P* < 0.05 and are statistically significant.

### PLR is an independent risk factor for in-hospital mortality

Univariate analysis revealed that hypertension; acute renal failure, pneumonia, sepsis, PLR, white blood cell count, serum sodium, blood glucose, prothrombin time, serum creatinine, serum calcium, and mechanical ventilation were significantly associated with in-hospital mortality. In the multivariable logistic regression analysis (see Table 3), when PLR was treated as a continuous variable, higher levels of PLR were associated with an increased risk of ICU mortality in patients with ischemic stroke [odds ratio (OR) = 1.14, 95% confidence interval (CI): 1.07–1.21, *P* < 0.001]. This conclusion remained consistent even after adjusting for confounding factors including hypertension; acute renal failure, pneumonia, sepsis, PLR, white blood cell count, serum sodium, blood glucose, prothrombin time, serum creatinine, serum calcium, and mechanical ventilation (Model 3, OR = 1.12, 95% CI: 1.04–1.19, *P* < 0.001). When PLR was considered as a categorical variable, the first group (PLR < 0.88) served as the reference category. The second group (0.88 ≤ PLR < 1.73) had an OR of 1.23 (95% CI: 0.71–2.15, *P* = 0.458), and the third group (PLR ≥ 1.73) had an OR of 3.36 (95% CI: 2.06–5.50, *P* < 0.001), both of which demonstrated a higher risk of in-hospital mortality. The OR values for PLR levels remained stable across the three models after adjustments in the multivariable model.

**TABLE 3:** Relationships between PLR levels and in-hospital mortality in different models.

Variables	Model 1		Model 2		Model 3	
	**OR (95% CI)**	***P***	**OR (95% CI)**	***P***	**OR (95% CI)**	***P***
PLR	1.14 (1.07 ∼ 1.21)	**<0.001**	1.10 (1.03 ∼ 1.17)	**0.006**	1.12 (1.04 ∼ 1.19)	**0.001**
PLR three						
PLR < 0.88	1.00 (reference)		1.00 (reference)		1.00 (reference)	
0.88 ≤ PLR < 1.73	1.23 (0.71 ∼ 2.15)	0.458	1.42 (0.78 ∼ 2.56)	0.251	1.55 (0.85 ∼ 2.83)	0.157
PLR ≥ 1.73	3.36 (2.06 ∼ 5.50)	**<0.001**	2.93 (1.72 ∼ 5.01)	**<0.001**	3.42 (1.97 ∼ 5.95)	**<0.001**

Bold values indicate *P* < 0.05 and are statistically significant.

### Non-linear relationship and threshold effect analysis

**FIGURE 2:**
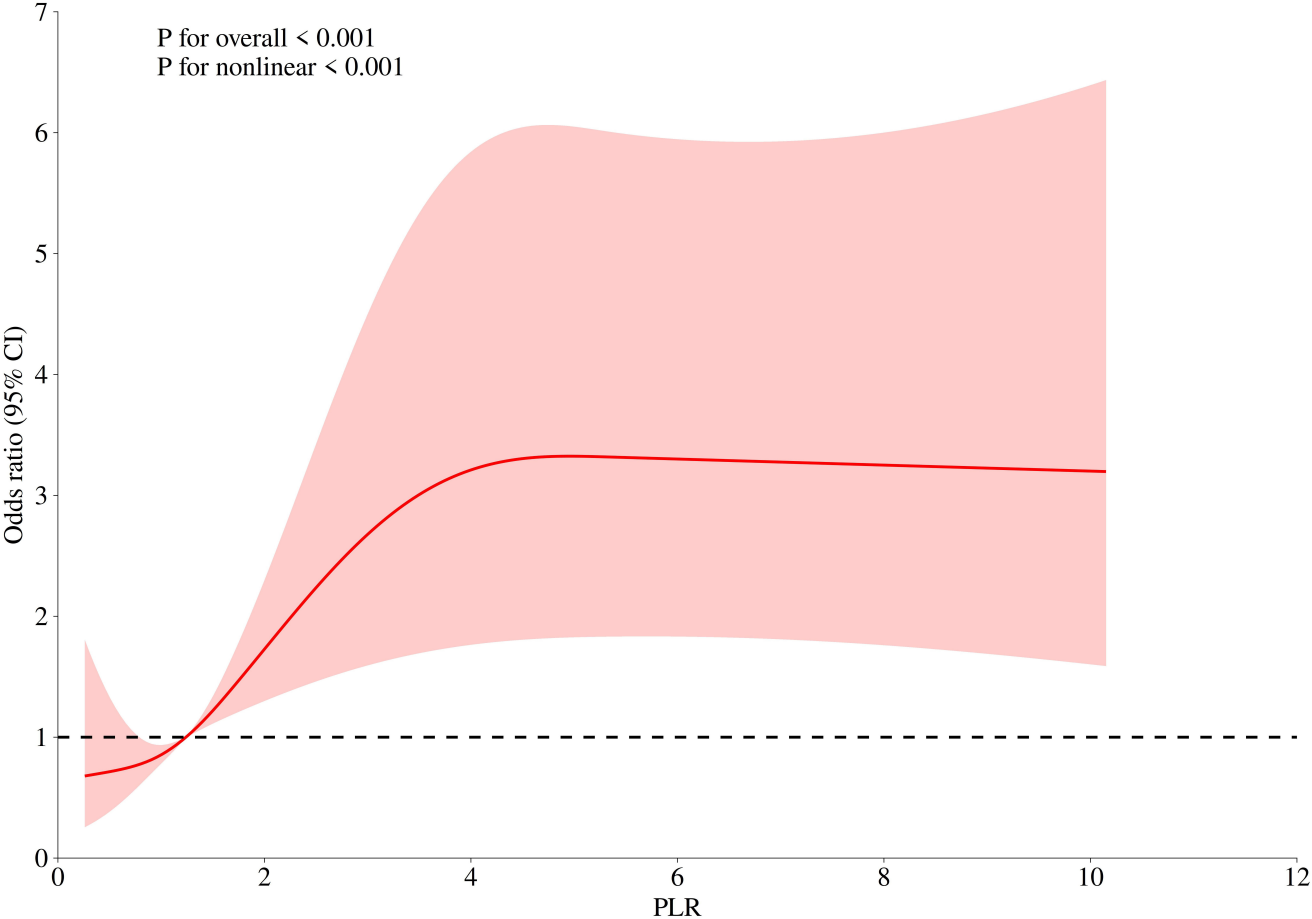
The non-linear relationship between PLR and in-hospital mortality in ICU patients with cerebral infarction (adjusted for all covariates as model 3).

We further explored the relationship between PLR levels and in-hospital mortality through multivariable-adjusted restricted cubic spline analysis. The non-linearity test showed a *P*-value of less than 0.05, revealing a J-shaped association, which indicated a non-linear relationship between PLR levels and mortality (Figure 2). After adjusting for confounding variables including hypertension (HT), acute renal failure (ARF), pneumonia, mechanical ventilation, sepsis, white blood cell count (WBC), sodium, total calcium, glucose, prothrombin time (PT), and creatinine, we used a single-slope model (Model 1) to show a significant positive correlation between PLR and in-hospital mortality. We then employed a likelihood ratio test to confirm the segmented model (Model 2) for threshold analysis. The results showed that there was a threshold effect in the association between PLR and in-hospital mortality (P for likelihood test < 0.001), meaning that the likelihood ratio test confirmed that the segmented model significantly improved the fit compared with the single-slope model. Overall, there was a positive association between PLR and in-hospital mortality [OR (95% CI): 1.12 (1.04–1.19)]. When PLR was less than 4.21, there was a positive association between PLR and in-hospital mortality [OR (95% CI): 1.56 (1.21–2.01)]. When PLR was greater than or equal to 4.21, no association was found between PLR and in-hospital mortality. In addition, we conducted a statistical analysis of PLR values for the 1,002 patients enrolled in the study and found that 916 patients had PLR values less than 4.21, accounting for 91.42%. Only 86 patients had PLR values greater than or equal to 4.21, accounting for 8.58%. This indicated that in this study sample, the vast majority of patients had PLR values below this threshold (see Table 4).

**TABLE 4:** Threshold analysis.

Outcome	Effect	*P*
Model 1 fitting model by standard linear regression	1.12 (1.04–1.19)	**0.001**
Model 2 fitting model by two-piecewise linear regression		
Inflection point	4.21	
PLR < 4.21	1.56 (1.21–2.01)	**<0.001**
PLR ≥ 4.21	0.97 (0.86–1.10)	0.637
P for likelihood test		**<0.001**

Bold values indicate *P* < 0.05 and are statistically significant.

### Subgroup analysis and forest plot

**FIGURE 3:**
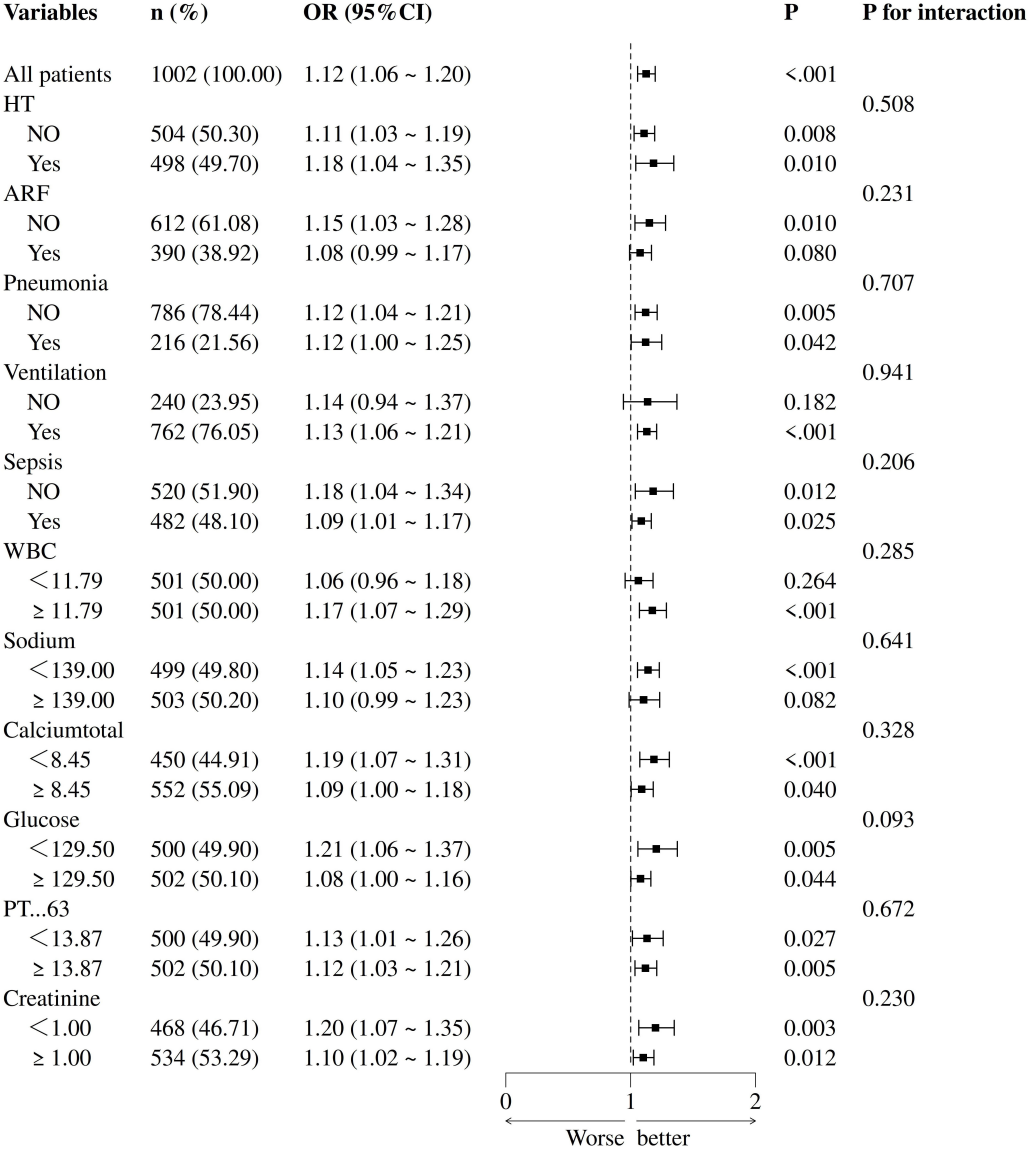
Forest plot of multivariate logistic regression subgroup analysis (adjusted for all covariates as model 3).

Logistic subgroup analysis revealed that elevated PLR levels were significantly associated with increased in-hospital mortality among ischemic stroke patients admitted to the ICU. Notably, this correlation was more pronounced and statistically significant in patients with acute renal failure, pneumonia, sepsis, those requiring mechanical ventilation, as well as individuals with high white blood cell counts, high serum sodium levels, high blood glucose values, high serum creatinine levels, low serum calcium, or prolonged prothrombin time. However, no statistically significant interaction effects were found across the subgroups (*P*-value for interaction > 0.05), indicating that demographic or clinical variables did not significantly modulate the association between PLR levels and in-hospital mortality (see Figure 3).

## Discussion

In this retrospective cohort study utilizing the MIMIC IV database, we clearly demonstrated that higher PLR levels were independently associated with an increased risk of in-hospital mortality. This relationship exhibited a non-linear pattern (with a non-linear *P*-value less than 0.05), meaning that within the range of PLR levels below 4.21, there was a positive correlation between PLR and in-hospital mortality. Subsequent subgroup analyses revealed similar association patterns. These findings hold significant clinical implications.

Regarding the relationship between PLR levels and the prognosis of stroke patients, evidence has been emerging continuously, but the results have not been consistent. Previous systematic reviews have found an association between PLR levels and mortality caused by ischemic cerebrovascular disease, but the nature of this association has not been elucidated (Sharma and Bhaskar, 2022). One study showed that an increased PLR was an independent predictor of hemorrhagic transformation and in-hospital mortality in acute atherosclerotic patients with acute ischemic stroke (Zhang et al., 2023). There have also been retrospective studies indicating that PLR levels were unrelated to the prognosis of ischemic stroke (Li, 2021; Ferro et al., 2021). The main controversy currently lies in whether PLR is associated with the prognosis of ischemic stroke and in determining the range of PLR. Multiple studies have reported that as PLR levels rise, the severity of ischemic stroke increases, and an observed positive correlation exists between infarct size and elevated PLR levels, but without specifying the range of PLR.

Our study confirmed that there was an independent association between the PLR level and the in-hospital mortality risk in ischemic stroke patients, presenting a “J”-shaped non-linear relationship. When the PLR was below 4.21, it was positively correlated with in-hospital mortality. However, when the PLR was above 4.21, no association between the PLR and in-hospital mortality was found. The mechanisms behind this may be as follows: Lymphocyte biological mechanisms: Lymphopenia and impaired immune function: (1) After stroke, the lymphocyte count usually decreases. This lymphopenia may weaken the body’s immune defense capabilities. For example, regulatory T cells (Tregs) have anti-inflammatory and neuroprotective effects after stroke, and a reduction in their numbers can lead to uncontrolled inflammatory responses, thereby increasing the risk of in-hospital mortality (Liesz et al., 2015). Impaired immune function may also result in secondary infections, which are a common cause of death in stroke patients (Zera and Buckwalter, 2020). (2) Exacerbation of inflammatory responses: An increase in the PLR may reflect an exacerbation of inflammatory responses. A decrease in lymphocytes may mean that the immune system is unable to effectively suppress inflammation, leading to elevated levels of inflammatory factors and further worsening of brain tissue damage (Yuan et al., 2023). The exacerbation of inflammatory responses can lead to complications such as brain edema and hemorrhagic transformation, increasing the in-hospital mortality rate (Yuan et al., 2025). (3) Saturation effect of inflammatory responses: At higher PLR levels, the inflammatory response may have reached a saturation point, where further increases in the PLR no longer significantly affect in-hospital mortality. This indicates that the intensity of the inflammatory response may have reached a critical threshold, beyond which its impact on prognosis is no longer significant (Yang et al., 2025; Zhai et al., 2021). Platelet-driven mechanisms: (1) Platelet activation and inflammatory responses: Platelets are activated after ischemic stroke and release inflammatory mediators such as platelet factor 4 (PF4) and interleukin-1β (IL-1β). These inflammatory mediators not only promote platelet aggregation but also activate local inflammatory responses (Fountain and Lappin, 2025). The inflammatory responses caused by platelet activation may further exacerbate brain tissue damage and increase the risk of in-hospital mortality. (2) Platelet function limit: At higher PLR levels, the activation and aggregation of platelets may have reached their limit, and further increases in the PLR no longer significantly affect in-hospital mortality. This suggests that the role of platelets in inflammatory responses and thrombosis may have reached a saturation point, beyond which their effects are no longer significant (Nascimento et al., 2024). Other factors: At higher PLR levels, other factors (such as comorbidities and individual differences) may have a more significant impact on in-hospital mortality, thereby masking the direct association between the PLR and in-hospital mortality (Hofmann et al., 2022). For example, patients with multiple chronic diseases may already have a higher risk of death, and changes in the PLR may no longer be the main determining factor in such cases.

These findings were consistent across subgroups with or without comorbidities (hypertension, acute renal failure, pneumonia, sepsis, mechanical ventilation) and laboratory indicators (white blood cell count, serum sodium, calcium, glucose, creatinine, prothrombin time). The potential mechanisms by which PLR levels affect the prognosis of ischemic stroke remain to be elucidated. The possible mechanisms currently include: (1) enhanced inflammatory response, where local brain tissue inflammation is activated due to ischemia and hypoxia after ischemic stroke, and platelets release inflammatory molecules that enhance the vascular inflammatory process (Zhang et al., 2019); (2) interaction between platelets and lymphocytes, where inflammatory mediators released by platelets during ischemic events may interact with lymphocytes, leading to a decrease in lymphocyte levels. This change may weaken the body’s immune protective function, further exacerbate the inflammatory response, and thereby increase the PLR value (Ludwig et al., 2022). (3) impact on reperfusion injury, where in patients with ischemic stroke undergoing endovascular therapy (EVT), elevated PLR levels are significantly associated with reperfusion failure (Lee et al., 2021). (4) relationship with hemorrhagic transformation, where studies have shown that elevated PLR levels are associated with an increased risk of hemorrhagic transformation (HT) in patients with ischemic stroke (Yang et al., 2021). Although research has revealed the association between PLR levels and the prognosis of ischemic stroke, the specific pathological mechanisms still require further investigation. Future research should focus more on the specific mechanisms of PLR in the inflammatory response and how to improve the prognosis of patients with ischemic stroke through interventions targeting PLR levels.

When PLR was below 4.21, the positive correlation between PLR and in-hospital mortality was more pronounced. This result indicates that at low PLR levels, an increase in PLR may be an important factor in increasing the risk of in-hospital mortality. This association may be related to the intensity of the inflammatory response (Hu et al., 2024). As an inflammatory marker, a lower level of PLR elevation may reflect an intensified inflammatory response, which in turn has an adverse effect on the patient’s prognosis. When the inflammatory response is more pronounced, the activation of platelets and the relative reduction of lymphocytes may jointly promote disease progression, thereby increasing the risk of in-hospital mortality (Oylumlu et al., 2015). However, when PLR was above 4.21, no significant association between PLR and in-hospital mortality was found. There may be several explanations for this result. First, it may indicate that at higher PLR levels, the intensity of the inflammatory response has reached a plateau, and further increases no longer significantly affect the risk of in-hospital mortality (Yang et al., 2025). Second, it may also suggest that at high PLR levels, other factors (such as individual differences among patients, the presence of comorbidities, etc.) have a more prominent impact on in-hospital mortality, thereby masking the direct association between PLR and in-hospital mortality (Hu et al., 2024). Therefore, we divided the enrolled patients into two groups based on whether their PLR values were less than 4.21 and conducted intergroup difference analyses. The results showed that patients with PLR ≥ 4.21 had a higher Charlson Comorbidity Index (*P* < 0.001), reflecting the presence of multiple chronic diseases in these patients and suggesting that comorbidities may play an important mediating role between the inflammatory response and in-hospital mortality. Moreover, the limited sample size may also have affected our ability to detect a significant association at higher PLR levels. In our study, we performed statistical analysis on the PLR values of 1,002 enrolled patients and found that only 8.58% (86 cases) had PLR values above 4.21. The relatively small sample size may have led to insufficient statistical power, making it difficult to accurately reflect the relationship between PLR and in-hospital mortality. This sample characteristic may have certain implications for the generalizability of the study results. In other studies, if the distribution of PLR in the sample is different, different association patterns may be observed. Therefore, future research needs to validate the relationship between PLR and in-hospital mortality in more diverse populations to determine the universality of these findings.

Despite this, our study still had certain limitations. First, as a retrospective cohort study, it is inherently subject to residual bias and unmeasured confounding factors; second, since the data were only from the MIMIC-IV database, this may affect the generalizability of the predictive model to other populations. Therefore, it is necessary to conduct large-scale, multicenter studies for external validation; third, the MIMIC database did not record patients’ pre-hospital medication use, so we were unable to assess the impact of this confounding factor in our study; fourth, the MIMIC-IV database lacked data on the National Institutes of Health Stroke Scale (NIHSS) scores, which hindered its use as a covariate indicating neurological deficits; fifth, our study did not determine patients’ rehabilitation status after discharge, which is still an important part of post-stroke functional recovery; sixth, blood samples were only collected within 24 h after symptom onset. Since continuous measurements were not taken, the dynamic changes in the platelet-to-lymphocyte ratio (PLR) and their association with the prognosis of acute ischemic stroke patients remain unclear. Lastly, when PLR ≥ 4.21, some samples do not fit this non-linear relationship. Future studies need to verify the relationship between PLR and in-hospital mortality in larger samples to ensure the generalizability and reliability of the results.

## Conclusion

Our study indicates that the PLR has the potential to serve as a predictor of in-hospital mortality for ischemic stroke patients in the ICU. We found a J-shaped relationship between the PLR and in-hospital death in ischemic stroke patients. PLR can help identify high-risk patients effectively. However, more evidence is still needed to support the clinical application of PLR.

## Data Availability

The raw data supporting the conclusions of this article will be made available by the authors, without undue reservation.
